# Distinct Differences on Neointima Formation in Immunodeficient and Humanized Mice after Carotid or Femoral Arterial Injury

**DOI:** 10.1038/srep35387

**Published:** 2016-10-19

**Authors:** Jill Moser, Joris van Ark, Marcory C. van Dijk, Dale L. Greiner, Leonard D. Shultz, Harry van Goor, Jan-Luuk Hillebrands

**Affiliations:** 1 Department of Pathology and Medical Biology-Pathology, University of Groningen, University Medical Center Groningen, Groningen, The Netherlands; 2 Department of Pathology, Rijnstate Hospital, Arnhem, The Netherlands; 3 Diabetes Center of Excellence, Program in Molecular Medicine, University of Massachusetts Medical School, Worcester, MA, USA; 4 The Jackson Laboratory, Bar Harbor, ME, USA

## Abstract

Percutaneous coronary intervention is widely adopted to treat patients with coronary artery disease. However, restenosis remains an unsolved clinical problem after vascular interventions. The role of the systemic and local immune response in the development of restenosis is not fully understood. Hence, the aim of the current study was to investigate the role of the human immune system on subsequent neointima formation elicited by vascular injury in a humanized mouse model. Immunodeficient NOD.Cg-*Prkdc*^*scid*^*IL2rg*^*tm1Wjl*^(NSG) mice were reconstituted with human (h)PBMCs immediately after both carotid wire and femoral cuff injury were induced in order to identify how differences in the severity of injury influenced endothelial regeneration, neointima formation, and homing of human inflammatory and progenitor cells. In contrast to non-reconstituted mice, hPBMC reconstitution reduced neointima formation after femoral cuff injury whereas hPBMCs promoted neointima formation after carotid wire injury 4 weeks after induction of injury. Neointimal endothelium and smooth muscle cells in the injured arteries were of mouse origin. Our results indicate that the immune system may differentially respond to arterial injury depending on the severity of injury, which may also be influenced by the intrinsic properties of the arteries themselves, resulting in either minimal or aggravated neointima formation.

Percutaneous coronary interventions (PCIs) are widely used to treat patients with severe coronary atherosclerotic disease. Although PCI is considered an effective and safe treatment modality to establish coronary revascularization, an important drawback is the development of restenosis of the treated artery^1^. Restenosis is characterized by re-narrowing of the artery due to the formation of an occlusive neointima. Neointimal lesions are characterized by smooth muscle cell (SMC) proliferation and extracellular matrix deposition^2^. Although the causative mechanisms underlying development of restenosis are not fully understood, systemic and local inflammation are considered important driving forces^1^,^2^,^3^. Both clinical and experimental data suggest that T cell recruitment into the treated arterial wall promotes neointima formation, however, contradictory results have also been reported. Immunodeficiency in experimental models was found to augment neointima formation after arterial injury in both immunodeficient mice and T cell-depleted or athymic rats^4^,^5^,^6^,^7^. In contrast, adoptive T cell transfer into immunodeficient *Rag1*^*null*^ mice reduced neointima formation; an effect which may be attributed to IFNγ^8^. More recent experimental evidence identified a role for CD8^+^ T cells in inhibiting neointima formation^7^. However, in contrast to the protective role of T cells and IFNγ on neointima formation, transfer of CD4^+^ T cells increased atherosclerosis in ApoE/immune deficient mice^9^, and IFNγ was shown to augment native atherosclerosis^10^ and graft atherosclerosis^11^. In addition to the role of T cells in the development of restenosis, the origin of SMCs within neointimal lesions is also under debate. Although it was generally believed that neointimal SMCs are derived from the adjacent tunica media similar to SMCs in atherosclerotic lesions^12^, in the last 15 years it has become evident that alpha-smooth muscle actin (αSMA) expressing neointimal cells derived from circulating progenitors can also contribute to vascular remodeling^13^,^14^. We previously demonstrated host-origin of neointimal SMCs in experimental transplant vasculopathy in rats^15^ and more recently in human renal allografts^16^. Direct evidence supporting the contribution of (bone marrow-derived) circulating αSMA expressing cells in the development of human restenosis is lacking. However, human atherosclerotic plaques were shown to contain bone marrow-derived αSMA expressing cells^17^. In line with this, human peripheral blood was shown to contain a population of smooth muscle progenitor cells^18^,^19^.

Much speculation therefore exists with respect to the precise roles of T cells in response to arterial injury which is complicated by the fact that researchers use diverse immunodeficiency models and different modes of arterial injury. Nevertheless, based on the available literature we hypothesized that the specific immune response to arterial injury depends on the method, and thereby severity, of arterial injury. Denudation wire-injury is considered a severe method to induce neointima formation. Here the endothelium is removed and the artery stretched due to the size of the guide-wire. The combination of denudation and arterial stretch results in robust neointima formation. The other model used in this study was the perivascular cuff method. Here the artery is sheathed with a polyethylene tube around the femoral artery. The tube is larger than the vessel so that the blood flow is not obstructed. The exact mechanism of neointima formation is currently unknown.

In this study we developed a humanized mouse model which would allow the investigation of: *1)* the involvement of human peripheral blood mononuclear cells (PBMCs) in the immune responses and subsequent neointima formation elicited by vascular injury, and *2)* the contribution of human PBMC-derived αSMA expressing cells to neointima formation in 2 widely-used models for neointima formation in mice.

## Results

### Neointima formation after single vs. dual injury in BL/6 mice

**Figure 1:**
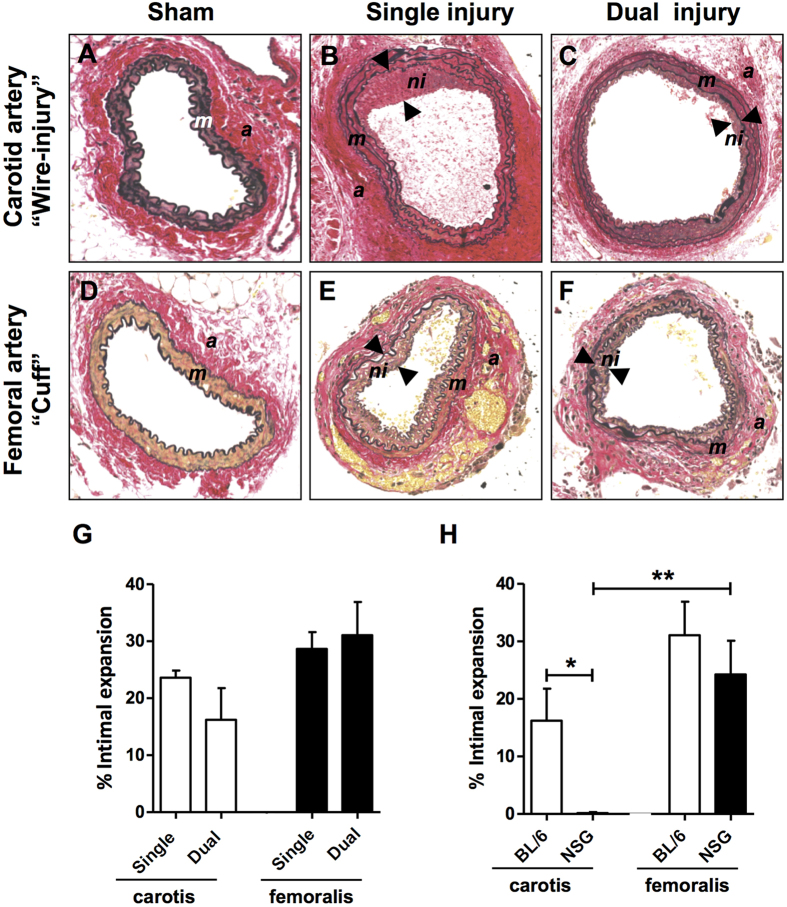
Neointima formation 4 weeks after dual vascular injury in BL/6 and NSG mice. Representative sections of carotid and femoral arteries from dual injured and sham-operated BL/6 mice stained with Verhoeff-van Gieson (**A–F**). Neointima is indicated as tissue in between arrowheads. (**A**) Sham-operated control carotid artery, (**B**) Carotid artery after single wire-denudation injury, (**C**) Carotid artery after wire denudation in a dual injured mouse, (**D**) Sham-operated control femoral artery, (**E**) Single collar-injured femoral artery, and (**F**) Collar-injured femoral artery in a dual injured mouse. Abbreviations: *a*: adventitia; *m*: media; *ni*: neointima. (**G**) Intimal expansion in arteries of single (n = 5) and dual (n = 5) injured BL/6 mice. No significant difference in intimal expansion was detected when comparing single versus dual injured mice. (**H**) Intimal expansion measured in the arteries of dual injured BL/6 (n = 5) and NSG (n = 9) mice. No significant difference was found in the amount of neointima observed between the femoral arteries of BL/6 and NSG mice, whereas intimal expansion in the carotid artery of NSG mice was significantly reduced when compared with BL/6 mice (*P < 0.05). In NSG mice, femoral arteries displayed significant intimal expansion compared with carotid arteries (**P = 0.01). Data are expressed as mean ± SEM. Original magnification (**A–F**): 200x.

Neointima formation may vary significantly depending on the type of vascular injury^20^,^21^, including the contribution of circulation-derived αSMA expressing cells to neointima formation^22^. Since we wanted to study the differential effects of arterial injury (*i.e.*, carotid wire versus femoral cuff injury) in NSG mice, we first had to validate the dual injury model and compare data with single injury. Both the carotid wire and femoral cuff injury model are widely used to study neointima formation, but to our knowledge these injuries have not been performed simultaneously in the same mouse. We carried out either single or dual injury in BL/6 mice which resulted in neointimal lesion formation 4 weeks after injury when compared to sham-operated mice (Fig. 1A–F). After single injury, the femoral cuff model appeared to induce slightly more neointima formation (expressed as % intimal expansion) when compared to carotid wire injury (23% *vs.* 28%, respectively, Fig. 1G). No significant differences in intimal expansion in femoral and carotid arteries were observed when comparing single injured and dual injured BL/6 mice (Fig. 1G). Similar to single injury, dual injury resulted in the development of an αSMA^+^ SMC-rich neointima (Fig. S2B,D). Thinning of the tunica media was observed in the femoral artery 4 weeks after cuff injury (Fig. S2D,E) yet the media of the carotid artery did not significantly differ after wire-injury (Fig. S2B,E). Masson’s trichrome staining identified collagen content in the media of the carotid artery after wire-injury (Fig. S2F). Yet, collagen deposition was not found in the neointima of either the carotid or femoral artery after injury (Fig. S2F,G). These data indicate that induction of dual injury does not influence the severity of neointima formation when compared to single injury.

### Neointima formation after dual injury in non-reconstituted NSG mice

Next, we investigated the role of immunodeficiency on neointima formation as this has not been studied in NSG mice. Dual arterial injury was carried out in NSG mice and the damaged arteries analyzed 4 weeks later. Unlike our findings in BL/6 mice, no neointima formation was observed in the wire-injured carotid arteries of NSG mice (P < 0.05 *vs.* BL/6 mice, Fig. 1H). In contrast, the femoral artery cuff which was placed in the same mouse did induce neointima formation (P = 0.01 *vs.* NSG carotid artery) to an extent similar to what was found in immunocompetent BL/6 mice (Fig. 1H). We therefore investigated the early response to dual vascular injury in NSG mice by examining the arteries of NSG mice 2 days after dual injury. Verhoeff-van Gieson staining (Fig. S3A), Masson’s trichrome staining (Fig. S4A–F) and immunofluorescent staining for αSMA (Fig. S3B,C) confirmed thinning of the femoral artery media as a result of cuff placement. In addition, the total number of cells was also reduced after cuff placement (Fig. S3D). In contrast, the media area of the carotid artery slightly increased (Fig. S3C) yet the total number of cells in the media of both the carotid artery was severely reduced 2 days after injury (Fig. S3D). In the femoral artery, a reduction in the number of cells present in the adventitia was also observed but the number of cells remained unchanged in the adventitia of the injured carotid artery (Fig. S3D). The reduction in cell numbers is likely a result of apoptosis early after injury. Staining for vWF confirmed endothelial staining in sham-operated arteries (Fig. S3e.1,e.3). In contrast, only a few vWF^+^ endothelial cells were present in injured carotid and femoral arteries confirming endothelial denudation 2 days after injury (Fig. S3e.2,e.4). At 2 days after injury we found thrombus formation in the carotid arteries of all mice (n = 4) (Fig. S3a.2,a.3) but not in the femoral artery arteries (Fig. S3a.5,a.6) which may be due to the differences between the vascular injury methods. In addition, it may also reflect the heterogeneity of the vasculature. Despite the susceptibility for thrombus formation in carotid arteries early after injury, this clearly did not result in neointima formation 4 weeks after injury (Fig. 1H).

### Generation of humanized NSG mice: engraftment of human PBMCs

**Figure 2:**
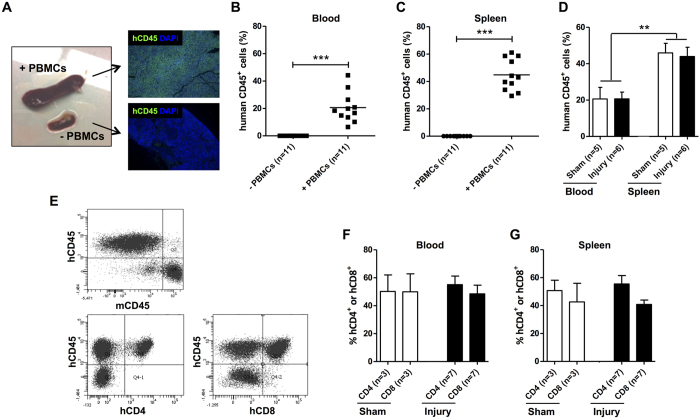
Engraftment of human PBMCs into NSG mice 4 weeks after reconstitution. NSG mice were reconstituted with 5 × 10^6^ human PBMCs by retro-orbital injection. Engrafted spleens showed marked splenomegaly and were repopulated with hCD45^+^ cells as revealed by immunofluorescent labeling (**A**). Engraftment of peripheral blood (**B**) and spleen (**C**) was evaluated by flowcytometry 4 weeks after reconstitution. Values of individual mice and median engraftment levels are shown (***P < 0.001). No differences in the engraftment of human CD45^+^ cells was observed in NSG mice which had first received arterial injury compared to sham-operated mice. Engraftment in spleen was significantly higher than in peripheral blood (**P < 0.01) (**D**). Representative dot plots of human PBMC-engrafted spleens are shown in panel (**E**). Human CD4^+^ and CD8^+^ cells were gated on live hCD45^+^ cells (**E**) and percentages given represent values of hCD4^+^ and hCD8^+^ cells within the hCD45^+^ population in peripheral blood (**F**) or spleen (**G**). The n-value reflects the number of mice included. Data are expressed as mean ± SEM.

To generate humanized NSG mice, we transferred 5 × 10^6^ human PBMCs isolated from the peripheral blood of 5 healthy male donors to NSG mice. PBMCs from each donor were used to reconstitute NSG mice that received arterial injury (n = 2) as well as sham mice (n = 2). NSG mice engrafted with human PBMCs are known to eventually develop symptoms of graft-*versus*-host disease (GvHD) depending on the number of PBMCs transferred. However, minimal GvHD occurs at one month when transferring 5 × 10^6^ PBMCs^23^. PBMCs were administered immediately after the dual arterial injury procedure was performed and mice sacrificed 28 days later. Reconstituted NSG mice showed marked splenomegaly (Fig. 2A). Immunofluorescent labeling for h(uman) CD45 revealed massive splenic repopulation with hCD45^+^ cells in engrafted NSG mice, whereas no hCD45^+^ were detected in non-reconstituted mice 4 weeks after administration. These data were supported by flow cytometric analyses performed on peripheral blood and spleen 4 weeks after reconstitution (Fig. 2B,C). As expected, no hCD45^+^ cells were detected in NSG mice that had not received human PBMCs. Higher levels of engraftment were observed in the spleen (44%) as compared to that achieved in the blood (21%) at 4 weeks (P < 0.01, Fig. 2B–D). Vascular injury did not alter the amount of engrafted hCD45^+^ cells, as similar levels were found in both sham-operated and injured mice (Fig. 2D). Almost all of the engrafted human cells in the blood and spleen were CD3^+^ (~99%) (data not shown). Within the population of hCD45^+^ cells the percentages of hCD4^+^ and hCD8^+^ cells were determined as depicted in Fig. 2E. The percentages of hCD4^+^ and hCD8^+^ in the blood and spleen were not significantly different in reconstituted NSG mice which had not received arterial injury compared to mice which had first received arterial injury (Fig. 2F,G). To determine GvHD in the PBMC-transferred NSG mice, we monitored for weight loss and performed haematological analyses at sacrifice. Although weight loss occurred shortly after arterial injury as a consequence of the surgical procedure, all mice had returned to their starting weight within a week and no differences were observed between PBMC-reconstituted and non-reconstituted mice (data not shown). A significantly higher number of white blood cells were detected in PBMC-reconstituted NSG mice (Fig. S5) which corresponds with the engraftment of human CD45^+^ cells (Fig. 2B). In NSG mice, red blood cell count, haemoglobin, haematocrit, and platelet counts were within the normal range and no differences were observed between reconstituted and non-reconstituted mice (Fig. S5B–E). These results confirm that the reconstituted NSG mice were not suffering from the detrimental effects of GvHD.

### Plasma levels of human and mouse cytokines in humanized NSG mice

**Figure 3:**
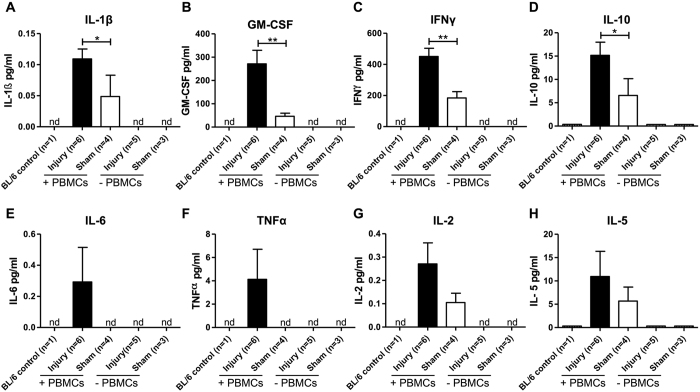
Plasma human cytokine levels in NSG mice reconstituted with (+) and without (−) 5 × 10^6^ human PBMCs, and with and without (sham) arterial injury. Plasma concentrations of human IL-1β (**A**), GM-CSF (**B**), IFNγ (**C**), IL-10 (**D**), IL-6 (**E**), TNFα (**F**), IL-2 (**G**) and IL-5 (**H**) were analyzed using an ultrasensitive cytokine bead array 4 weeks after PBMC reconstitution and arterial injury. Significant differences between injured and sham-operated reconstituted mice were found for IL-1β, GM-CSF, IFNγ and IL-10. The n-value reflects the number of mice included. Data are expressed as mean ± SEM. *P < 0.05, **P < 0.01. (nd =  not detectable; ns =  not significant).

In order to analyze whether dual vascular injury modulates systemic human PBMC cytokine production in humanized mice which may influence neointima formation, plasma samples were analyzed for cytokine levels using an ultra-sensitive 10-plex multiplex assay capable of detecting very low levels of human inflammatory cytokines. Plasma from both BL/6 mice and non-reconstituted (−PBMCs) NSG mice were used as controls. These control mice had no detectable levels of the human inflammatory cytokines analyzed (Fig. 3A–H). However, low levels of the human cytokines IL-1β (A), GM-CSF (B), IFNγ (C), IL-10 (D), IL-2 (G), and IL-5 (H) were measured in the plasma of mice reconstituted with human PBMCs (Fig. 3, white bars). Significantly elevated levels of IL-1β (P < 0.05, A), GM-CSF (P < 0.01, B, IFNγ (P < 0.01, C), and IL-10 (P < 0.05, D) were observed in PBMC-engrafted mice (compared with non-reconstituted mice) 4 weeks after receiving dual arterial injury (Fig. 3, black bars). Although not significant, increased plasma levels of human IL-2 (G), IL-5 (H), IL-6 (E) and TNFα (F) were also observed in reconstituted mice which had received dual arterial injury compared to uninjured reconstituted mice (Fig. 3). Inflammatory cytokines of mouse origin (IL-1β, IL-5 and IL-6) were not detected in the plasma of (non-) reconstituted NSG mice with or without arterial injury (data not shown).

### Neointima formation in dual injured PBMC-engrafted and non-reconstituted NSG mice

**Figure 4:**
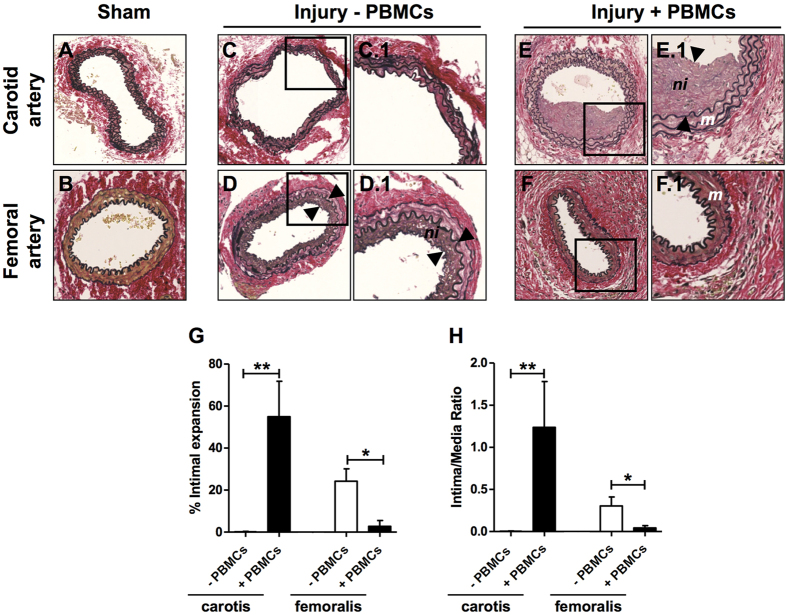
Neointima formation 4 weeks after dual vascular injury in NSG mice reconstituted with or without human PBMCs. Representative sections of carotid and femoral arteries from dual injured and sham-operated mice stained with Verhoeff-van Gieson. (**A**) Sham- operated control carotid artery, (**B**) Sham-operated control femoral artery, (**C**) Carotid artery after wire injury in a non-reconstituted NSG mouse, (**D**) Femoral artery after cuff injury in a non-reconstituted NSG mouse, (**E**) Carotid artery after wire injury in a human PBMC-reconstituted NSG mouse, (**F**) Femoral artery after cuff injury in a human PBMC-reconstituted NSG mouse. Squares indicate areas shown at higher-power magnification of the respective cross-sections (C.1, D.1, E.1, F.1). Neointima is indicated as tissue in between arrowheads. Neointima formation in the carotid and femoral arteries of dual injured NSG mice reconstituted with (n = 6) and without (n = 9) human PBMCs was expressed as % intimal expansion (**G**) and intima/media ratio (**H**). *P < 0.05, **P < 0.01. Data are expressed as mean ± SEM. Abbreviations: *m*: media; *ni*: neointima; PBMCs: human peripheral blood mononuclear cells. Original magnification (**A–F**): 200x.

We next examined the effect of human PBMC reconstitution in NSG mice on neointima formation after dual arterial injury. Significant neointima formation was found in the carotid artery of reconstituted NSG mice four weeks after arterial injury yet no neointimal lesions were observed in carotid arteries of NSG mice without human cell reconstitution (P < 0.01 *vs.* non-reconstituted NSG, Fig. 4A,C,E,G). In contrast, neointima formation was significantly reduced in the femoral arteries of reconstituted NSG mice when compared with non-reconstituted mice (P < 0.05, Fig. 3B,D,F,G). Similar results were obtained when expressing data as Intima/Media ratio (Fig. 4H).

### Smooth muscle cell phenotypic modulation after arterial injury

**Figure 5:**
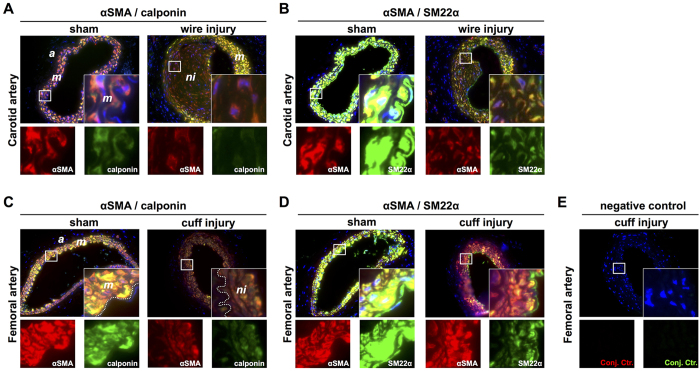
Neointimal cells in the injured carotid (PBMC-engrafted NSG mice) and femoral (non-PBMC-engrafted NSG mice) artery express αSMA, calponin and SM22α. Sham-operated (non-injured) and wire-injured carotid arteries stained for αSMA/calponin (**A**) and αSMA/SM22α (**B**). Sham-operated and cuff-injured femoral arteries stained for αSMA/calponin (**C**) and αSMA/SM22α (**D**). As negative control, cuff-injured femoral arteries were incubated according the protocol but with omitting primary antibody incubation (**E**). Insets and single stainings show high power magnifications of the boxed regions. Acquired photomicrographs were converted into pseudocolored images and merged using ImageJ 1.47v (http://imagej.nih.gov/ij). Abbreviations: *a*: adeventitia; *m*: media; *ni*: neointima.

In order to determine the SMC phenotype of neointimal cells in injured carotid and femoral arteries we performed immunofluorescent double labeling for αSMA/calponin and αSMA/SM22α. Non-injured carotid and femoral arteries were used as positive controls since differentiated medial SMCs within these arteries are known to express αSMA, SM22α and calponin (Fig. 5A–D, left panels). Medial SMCs in injured arteries also expressed all three SMC markers (Fig. 5A–D, right panels). In contrast, neointimal lesions in both the carotid and femoral arteries were characterized by low αSMA expression accompanied with reduced expression of SM22α and calponin supporting a de-differentiated proliferative SMC phenotype. Production of excessive connective tissue as a result of SMC phenotypic modulation from the contractile-type to the de-differentiated synthetic-type is proposed to play a major role in the response to vascular injury. Trichrome staining (Martius Scarlet Blue [MSB] revealed minimal collagen content in the media of sham-operated carotid (Fig. S4A,G) and femoral arteries (Fig. S4D,J). In contrast, MSB staining revealed extensive collagen deposition in the neointima of carotid arteries (Fig. S4I) and in the media of femoral arteries (Fig. S4L) of reconstituted mice 4 weeks after injury. In immunodeficient mice, collagen deposition in the neointima was minimal after femoral artery cuff injury (Fig. S4K) and was absent in the media of the carotid artery after wire-injury (Fig. S4H). Hence, SMC phenotypic modulation was independent of the immune status of the mice. However, in contrast to immunocompromised (no PBMCs) mice, collagen deposition found in the neointima and media of injured arteries appeared to be more extensive in the arteries of reconstituted mice.

### Human leukocyte infiltration in dual injured PBMC–engrafted NSG mice

**Figure 6:**
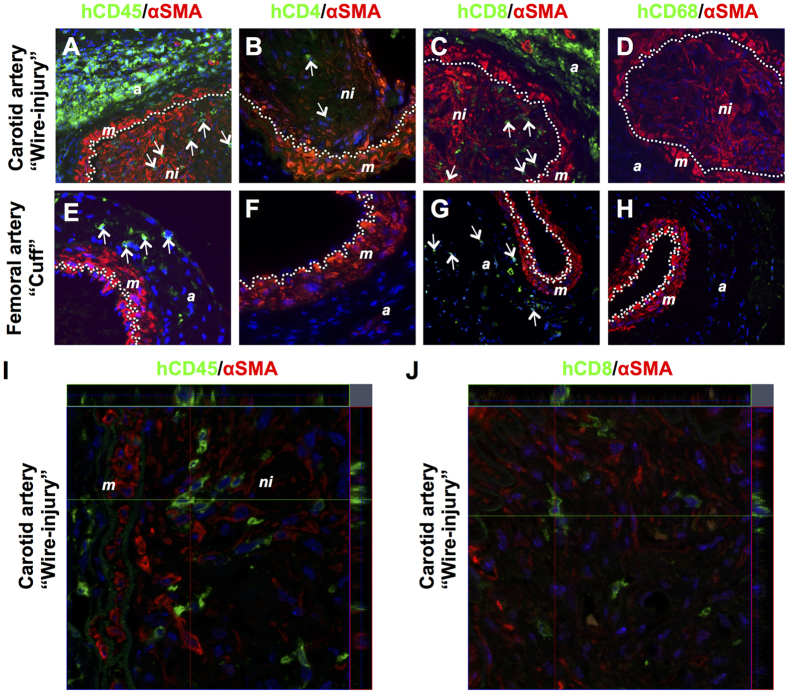
Presence of human PBMC-derived cells within injured carotid (**A–D**) and femoral (**E–H**) arteries in NSG mice 4 weeks after reconstitution with 5 × 10^6^ human PBMCs. Sections were immunofluorescently stained with αSMA (in red) and specific antibodies (in green) recognizing human CD45 (**A,E**), human CD4 (**B,F**), human CD8 (**C,G**) and human CD68 (**D,H**). Representative images of arterial staining from 3 different mice per group. Specificity controls for the antibodies used are shown in Figs S6 and S7A. Nuclei were stained with DAPI (in blue). Dotted line indicates the internal elastic lamina. Arrows indicate positively stained human leukocytes. Original magnification: 400x. Confocal microscopy ortho images hCD45/αSMA (I) or hCD8/αSMA (**J**) on wire-injured femoral arteries. Abbreviations: *a*: adventitia; *m*: media; *ni*: neointima.

In order to determine if the differences in neointima formation in injured femoral and carotid arteries in PBMC-reconstituted and non-reconstituted NSG mice were associated with human leukocyte infiltration, double immunofluorescent labeling was performed on arterial sections using human-specific antibodies against pan-leukocytes (hCD45), T cell subsets (hCD4 and hCD8) and macrophages (hCD68) combined with αSMA. The species specificity of the human-specific antibodies was confirmed by staining spleen sections of PBMC-reconstituted and non-reconstituted NSG mice as well as human appendix (positive control) with the appropriate antibodies using immunohistochemistry (Fig. S6) and immunofluorescence (Fig. S7). Staining of injured carotid artery sections revealed the presence of human PBMC-derived hCD45^+^ leukocytes in large numbers in the adventitia but also within the neointima 4 weeks after injury (Fig. 6A). The majority of these hCD45^+^ cells appeared to be hCD8^+^ (Fig. 6C), whereas only few hCD4^+^ cells were observed within the neontima (Fig. 6B). Moreover, no human-derived CD68^+^ macrophages were found within the neointima, media or adventitia of the injured carotid artery (Fig. 6D). In PBMC-reconstituted mice virtually no neointimal lesions were present within the femoral arteries of the same dual injured mice. In the adventitia of these arteries hCD45^+^ were present (Fig. 6E) which were found to be primarily hCD8^+^ cells (Fig. 6G). No hCD4^+^ or hCD68^+^ cells were found (Fig. 6F,H).

### No human-PBMC derived SMCs and ECs in dual injured PBMC-engrafted NSG mice

Neointimal SMCs expressing αSMA may be in part derived from circulating presumed myeloid, progenitor cells. To analyze whether in our humanized mouse model PBMC-derived αSMA expressing neointimal cells were present, double labeling was performed for hCD45 (as well as hCD4, hCD8 and hCD68) and αSMA. Although hCD45^+^ cells were present in the αSMA^+^ neointima, clearly no CD45^+^αSMA^+^ double positive cells were detected (Fig. 6A). The same was true for the other markers included (hCD4, hCD8 and hCD68, Fig. 6B–D). In addition, high resolution confocal microscopy revealed no co-expression of hCD45 or hCD8 with SMA^+^ neointimal SMCs (Fig. 6I,J). These data suggest that the αSMA^+^ SMCs in carotid neointimal lesions in humanized NSG mice are not derived from human PBMCs.

**Figure 7:**
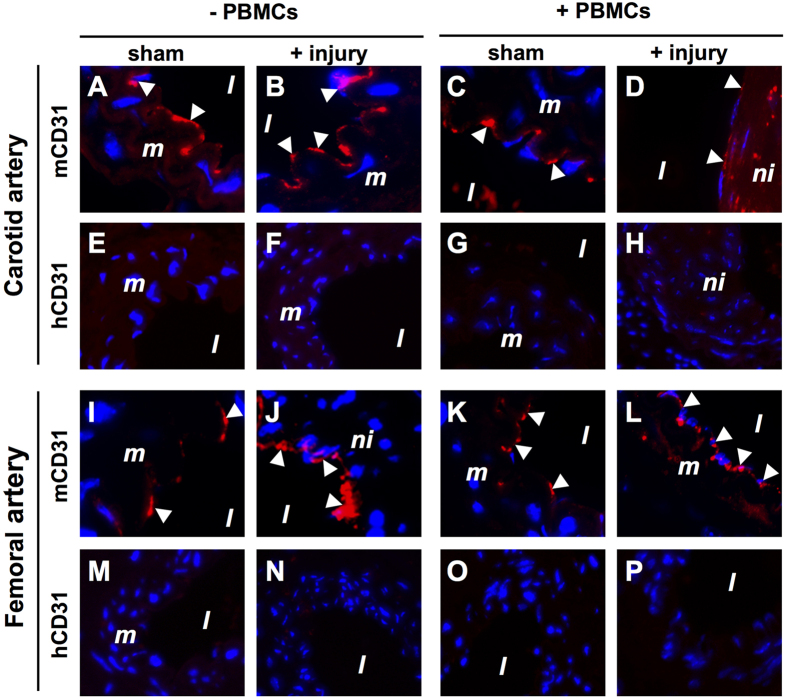
No re-endothelialization of carotid (**A–H**) and femoral (**I–P**) arteries with human PBMC-derived CD31+ endothelial cells in NSG mice 4 weeks after reconstitution with 5 × 10^6^ human PBMCs. Sections from sham-operated and injured carotid and femoral arteries obtained from both human PBMC reconstituted (+PBMCs) and non-reconstituted (−PBMCs) NSG mice were included in this analysis. Sections were immunofluorescently stained with anti-human CD31 (hCD31) and anti-mouse CD31 (mCD31) species-specific antibodies. Representative images of arterial staining from 3 different mice per group. Specificity controls for the hCD31 antibody is shown in Fig. S6B. Nuclei were stained with DAPI (blue). Arrowheads indicate positively stained CD31^+^ endothelial cells. Abbreviations: *l*: lumen; *m*: media; *ni*: neointima; PBMCs: human peripheral blood mononuclear cells. Original magnification: 400x.

In Fig. S3 we showed endothelial denudation in both carotid and femoral arteries early after injury. This observation raised the question whether re-endothelialization is mediated (in part) by endothelial progenitor cells which are known to reside within the PBMC fraction. To address this question, species-specific immunofluorescence was performed using antibodies directed against hCD31 and mCD31. The specificity of the hCD31 antibody was confirmed on human venous tissue (Fig. S7B). In sham-operated carotid and femoral arteries, both in non-reconstituted (Fig. 7A,I) and PBMC-reconstituted (Fig. 7C,K) NSG mice the endothelium was of mouse origin. Also 4 weeks after injury, both in non-reconstituted (Fig. 7B,J) and PBMC-reconstituted (Fig. 7D,L) mice the endothelium was of mouse origin. Human PBMC-derived ECs were never detected in carotid (Fig. 7E–H) and femoral (Fig. 7M–P) arteries. Similar to neointimal SMCs, these data indicate that ECs in injured arteries in humanized NSG mice are not derived from human PBMCs.

### Neointima formation in the femoral artery after wire-injury

**Figure 8:**
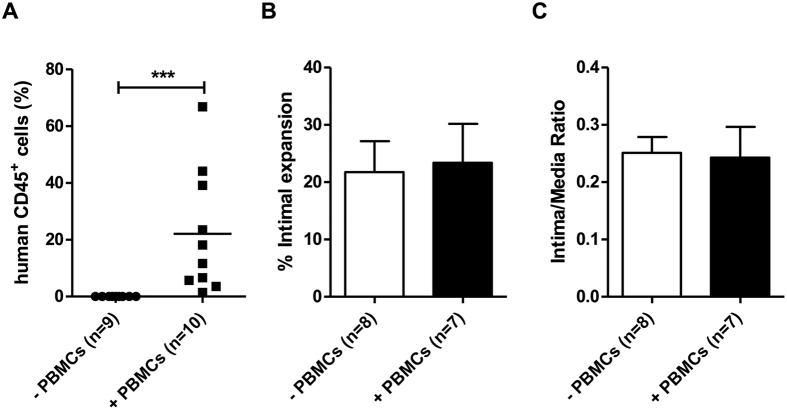
Neointima formation 4 weeks after femoral artery wire injury in NSG mice reconstituted with or without human PBMCs. (**A**) NSG mice reconstituted with 5 × 10^6^ hPBMCs and engraftment of peripheral blood was evaluated by flow cytometry 4 weeks after reconstitution. (**B**) Neointima formation in the wire injured femoral arteries of NSG mice reconstituted with (n = 7) and without (n = 8) hPBMCs was expressed as % intimal expansion and (**C**) intima/media ratio ***P < 0.001. Data are expressed as mean ± SEM.

To determine whether the type of artery or the method of inducing injury (wire vs cuff) is important we also performed wire-injury in the femoral artery of NSG mice with or without PBMC reconstitution. Four weeks after surgery and reconstitution the level of chimerism in the peripheral blood was about 22% (Fig. 8A) consistent with our other studies (Fig. 2B). In contrast to the carotid artery, femoral artery wire injury resulted in neointima formation already without reconstitution. Furthermore, reconstitution with hPBMCs did not attenuate neointima formation in wire-injured femoral arteries (Fig. 8B,C). Hence, neointima formation in the femoral artery of immunocompromised mice is not dependent on the type of vascular injury since both the cuff and wire injury resulted in around 20% neointimal expansion.

## Discussion

The mechanisms underlying restenosis development following PCI remain unclear. As a consequence additional experimental models are needed to investigate the interaction between the (human) immune system and the vascular wall in neointima formation. In the current study we developed a humanized mouse model based on the immunodeficient NSG mouse strain. NSG mice lack functional T cells, B cells, and NK cells. Moreover NSG mice have functional defects in macrophages, and dendritic cells and have no hemolytic complement^24^,^25^. Reconstitution of NSG mice enabled us to investigate the involvement of human PBMCs in the immune responses and subsequent neointima formation elicited by vascular injury, and, secondly, to determine the contribution of human PBMC-derived αSMA expressing cells to neointima formation. The severity of neointima formation and the contribution of circulating cells to neointimal lesions varies depending on the type of vascular injury^20^,^21^,^22^,^26^. We therefore applied two commonly used experimental methods of vascular injury, *i.e.* carotid artery wire injury and femoral artery cuff injury, together in the same mouse to study the effect of human PBMC reconstitution on neointima formation. Wire injury results in endothelial denudation and is known to disrupt the internal elastic lamina and the media whereas placement of a polyethylene cuff loosely over the femoral artery stimulates slow sloughing of the endothelium within 1–2 days after placement^27^. We hypothesized that differences in the severity of injury and the exposed denuded vasculature would influence endothelial regeneration, homing of inflammatory and progenitor cells, and the proliferation of medial SMCs.

Dual injury in immunocompetent BL/6 mice resulted in neointima formation in both the carotid and femoral arteries. However, in non-reconstituted NSG mice significant neointima formation was observed only in the femoral artery 4 weeks after cuff placement. This observation is in line with previous studies in immunodeficient *Rag1*^*−*/*−*^ mice also showing intimal hyperplasia after femoral artery injury^7^,^8^. In contrast, we surprisingly found no neointima formation in the carotid artery of the same non-reconstituted NSG mice. We had expected substantial neointima formation after wire-induced injury in the carotid artery since this model is thought to be more severe when compared to the perivascular cuff model of the femoral artery^28^. From these results we concluded that immunodeficiency protects against neointima formation which is dependent on the type of injury. To our knowledge, this is the first study that describes the differential (both protective and stimulatory) effects of immunodeficiency on simultaneous carotid wire-injury and femoral artery cuff-injury induced neointima formation in mice.

We next examined the effect of reconstitution on neointima formation after dual arterial injury in NSG mice. High levels of human lymphocyte engraftment with primarily CD3^+^ T cells were observed in peripheral blood and spleen 4 weeks after reconstitution. In contrast to what we observed in NSG mice without reconstitution, we found that the femoral artery in humanized mice developed no neointima 4 weeks after arterial injury whereas the carotid artery developed severe neointimal lesions. These results indicate that human cell reconstitution, which was predominantly composed of T cells, protects against neointima formation in the femoral artery after cuff injury but not after wire-induced injury, and promotes neointima formation in the carotid artery after wire-induced injury.

Since femoral and carotid arteries are diametrically different (*i.e.* femoral arteries are approximately 3 times smaller in diameter), the severity of vascular injury induced by the guide wire is most likely different (*i.e.* increased damage in femoral artery), and consequently the remodeling response. Carotid arteries have 3 layers of elastin and 2 layers of SMCs, whereas the femoral artery has 2 layers of elastin and only one layer of SMCs. In addition to the amount of media (SMCs), vessel elasticity, hemodynamic responses, the basement membrane and other factors such as residual endothelium play important roles in driving the response to arterial injury. Based on our results we could not discriminate whether the type of artery or the method of inducing injury is the most important factor in driving neointima formation in immunodeficient NSG mice. We therefore also performed femoral artery wire injury in NSG mice with or without PBMC reconstitution. Wire-injured femoral arteries developed neointimal lesions to a similar extent as that observed in femoral cuff injured arteries (approximately 20% intimal expansion) in NSG mice without reconstitution. These results suggest that neointima formation in the femoral artery is not dependent on the type of vascular injury in immunocompromised mice. Moreover, because of the severity of wire-injury (denudation and stretching) in the femoral artery we speculate that occlusion may not be able exceed more than 20% intimal expansion which is likely attributed to an intrinsic property of the femoral artery itself. Besides, the carotid artery is composed of a larger SMC-rich media compared to the femoral artery which may explain why the carotid artery has more neointima formation compared to the femoral artery after injury. In reconstituted NSG mice, wire-injury of the femoral artery did not attenuate neointima formation (20% intimal expansion) compared to femoral cuff arteries with only 4% intimal expansion. These results are in line with substantial neointima formation observed in the carotid artery after wire injury in reconstituted NSG mice. Likely the immediate and complete removal of the endothelial layer in the femoral artery after wire-injury promotes the recruitment of the hPBMCs which were systemically administered to the mice immediately after the surgical procedure. These results suggest that direct recruitment of hPBMCs to the injured arterial wall has detrimental effects (i.e. enhancing or loss of protective effects) which drive neointima formation in the denuded arteries. In contrast, EC removal is slower by the perivascular cuff which likely results in the differential effects of hPBMCs on neointima formation seen in our study. Together, these results importantly highlight the complexity of neointimal lesion formation and the role of T cells in this process and suggest that the immune system may differentially respond to arterial injury depending on the method of injury, which may also be influenced by the intrinsic properties of the arteries themselves.

After arterial injury apoptosis may play an important role on limiting vascular SMC proliferation and corresponding neointima formation. However, excessive apoptosis can lead to necrosis and inflammation. In a rat arterial injury model, 2 waves of apoptosis were identified, early from 30 min until 6 hours after injury initiation, and late apoptosis around 2 weeks after arterial injury^29^. Early apoptosis can lead to inflammation which is thought to contribute to the remodelling process. We found severely reduced numbers of SMCs in the media of the carotid and femoral arteries at 2 days after injury. In addition, adventitial cells were also reduced. This reduction in the amount of cells is likely due to apoptosis.

In our study, no human PBMC-derived neointimal SMCs were detected, indicating that the αSMA^+^ neointimal SMCs were of mouse origin. This is remarkable as it was previously shown that endovascular wire-injury in mice results in neointimal lesions containing significant numbers (~27%) of bone marrow-derived αSMA^+^ cells^22^. A possible explanation might be that engrafted PBMCs in NSG mice are primarily T cells whereas it has been shown that circulating smooth muscle progenitor cells probably reside within the myeloid population^18^,^19^. Moreover, no human PBMC-derived ECs were detected in the injured femoral and carotid arteries. These data are in line with a report by Hagensen and colleagues in which it was shown that circulating endothelial progenitor cells do not contribute to endothelial regeneration after murine arterial injury^30^.

Immune cells play a crucial role in the formation of neointimal lesions by inducing the production of cytokines and chemokines that may affect EC and SMC survival, proliferation, migration and activation. As expected, we were unable to detect cytokines of mouse origin in any of the experimental NSG groups since these mice have impaired cytokine signaling. In contrast, 4 weeks after reconstitution non-injured NSG mice had basal levels of several human cytokines, yet mice that had received dual arterial injury had significantly higher levels of IL-1β, GM-CSF, IFNγ and IL-10. These data indicate that vascular injury triggered a functional cytokine release response in humanized mice. The contribution of these cytokines to neointima formation is not fully elucidated but manipulation of cytokines is a potential therapeutic option for the prevention or treatment of restenosis. This is also supported by clinical data obtained with sirolimus-eluting stents which significantly reduced in stent restenosis^31^. Cytokines such as IFNγ are thought to increase pro-inflammatory activity following coronary intervention and might therefore be related to the long-term complications after intervention^32^,^33^. Likewise, GM-CSF has been implicated in atherogenesis^34^ and has been shown to have both pro- and anti-atherogenic properties^35^ whereas GM-CSF deficiency in mice was shown to delay neointima formation after arterial injury^36^. Similarly, IL-10 is an anti-inflammatory cytokine with anti-atherogenic potential which has been shown to inhibit neointima formation after femoral artery injury in hypercholesterolemic mice^37^,^38^,^39^ and rabbits^40^. This IL-10-induced attenuation of neointima formation was attributed to its powerful inhibitory effects on the activation and growth of SMCs. Based on these published data we suspect that the increased cytokine levels, as detected in engrafted NSG mice after injury, may have had a direct effect on SMC behaviour and resulting neointima formation. However, a number of species-specific factors in mice are not cross-reactive with human cells and vice-versa. Therefore, the pro- and anti-inflammatory factors of human origin measured in the plasma of our mice may have had no influence on the neointima formation observed in this study. Human GM-CSF and IFNγ are known to be only biologically active on human cells^41^,^42^ and therefore probably have little influence on neointimal lesion formation after dual arterial injury in mice. However, human IL-10 does have cross-species activity and is known to be active on mouse cells^43^,^44^. We therefore presume that the IL-10 measured in our mice would have had an influence on the neointima formation observed in the current study and may be protecting against neointima formation after femoral artery cuff injury as observed in previous studies^36^. However, further studies are required to confirm if this is indeed the case.

Animal models of restenosis provide a tool for investigating the pathophysiological mechanisms as well as translational approaches to vascular interventions. Several murine experimental models exist, carotid and femoral artery wire injury are widely accepted as the most appropriate techniques in studies of post-angioplasty restenosis because it closely resembles the angioplasty procedure that injures both the endothelium and vessel wall driving neointima formation. The perivascular cuff method does not exactly mimic what happens in humans undergoing percutaneous coronary interventions but is widely used in the literature as a method to understand neointima formation. Moreover, it may be adopted as a model system for testing the perivascular delivery of therapeutic compounds in order to inhibit restenosis. Many conclusions are drawn from mouse models of arterial injury which have contributed to our general understanding of the mechanisms promoting neointima formation. However, whether these translate directly to the human situation is unclear. Our findings emphasize the importance of using the most accurate experimental model when investigating mechanisms of neointima formation and preclinical intervention studies.

Most studies to date described the effects of the normal immune system on neointima formation although it is known that immune cell function might be impaired in subjects at increased cardiovascular risk. A strength of the humanized mouse model described here is that this model can be adopted to understand the immune responses of vascular injury in populations which have a high risk of developing restenosis, e.g. diabetes^45^,^46^. Recent data indicate that immune and progenitor cells from type 2 diabetic subjects are naturally skewed towards pro-inflammatory subsets that likely promote chronic inflammation^46^. Understanding the unique aspects of T cells from diabetic patients as well as understanding their influence on neointima formation is essential in order to develop pharmaceutical agents to target restenosis.

In conclusion, we have developed a humanized NSG dual vascular injury model which allows us to study the contribution of the T cell system on neointima formation. We propose that the immune system differentially responds to arterial injury depending on the severity of injury which may also be influenced by vascular heterogeneity.

## Methods

### C57BL/6 and NOD.Cg-Prkdc^scid^IL2rg^tm1Wjl^ mice

Male 10-week-old C57BL/6 (BL/6) mice were purchased from Harlan (Horst, The Netherlands), housed under standard housing conditions and given free access to food and water. Male 10-week-old NOD.Cg-*Prkdc*^scid^*IL2rg*^*tm1Wjl*^ (NSG, Jackson Laboratory, Bar Harbor, ME) were housed in a specific pathogen-free facility in micro-isolator cages and given free access to autoclaved food and water. All experimental procedures were performed according to European Commission guidelines and Dutch laws and were approved by the animal ethics committee of the University of Groningen.

### Mouse carotid wire injury and femoral cuff injury models

In this study two different types of vascular injury were induced in the same BL/6 or NSG mouse. Both left common carotid artery (wire-injury) and right femoral artery (femoral cuff injury) of each mouse were injured to induce neointima formation. In contrast to carotid wire-injury, in the femoral cuff model the endothelial cells are not directly manipulated or removed. Mice were anesthetized with 2% isoflurane/O_2_ followed by carotid or femoral artery endothelial denudation and/or femoral artery cuffing. Briefly, the left carotid artery and its muscular branch or the left femoral artery was exposed, and a 0.014′ (0.36 mm) diameter angioplasty guide-wire (Cook Medical Europe Ltd., Limerick, Ireland) introduced into the arterial lumen and pulled back three times. Removal of the endothelium was confirmed by *in vivo* Evan’s blue staining in injured arteries compared with uninjured arteries (data not shown). For induction of cuff femoral artery injury, a non-constrictive polyethylene cuff (0.4 mm inner diameter, 0.8 mm outer diameter, length 2 mm, Portex, Kent, UK) was loosely placed around the right femoral artery. Contra-lateral carotid and femoral arteries as well as sham-operated mice were used as controls. Mice were euthanized at two days or four weeks after injury by deep anesthesia (5% isoflurane/O_2_) and exsanguination. Spleen and peripheral blood (collected in EDTA vacutainers (BD Biosciences, Franklin Lakes, NJ, USA)) were obtained, plasma was isolated and stored at −80 °C until use. After subsequent *in situ* cardiac perfusion with zinc fixative^47^, arteries were collected for histology. Haematological analyses were performed using the Poch-100i haematology analyser (Sysmex Nederland, Etten-Leur, the Netherlands) according to the manufacturer’s instructions.

### Isolation of human PBMCs for engraftment

Human PBMCs were obtained from five healthy male volunteers (average age 26 years) under signed informed consent in accordance with the 1964 Declaration of Helsinki (revised October 2008, Seoul) and approval from the Medical Ethical Committee of the University Medical Center Groningen. Whole blood samples were obtained by venipuncture and collected in EDTA Vacutainers. PBMCs were purified by Ficoll-Paque (GE Healthcare Europe GmbH, Diegem, Belgium) density centrifugation and resuspended in Hank’s buffer solution for retro-orbital injection into NSG mice at a cell dose of 5 × 10^6^ cells (in 200 μl). NSG mice were reconstituted with the human PBMCs immediately after the dual injury surgical procedure. The following experimental groups were included: (i) untreated NSG mice, (ii) NSG mice given 5 × 10^6^ PBMCs 4 weeks prior to analyses, (iii) NSG mice given 5 × 10^6^ PBMCs and dual arterial injury 4 weeks prior to analyses, and (iv) NSG mice which received only dual arterial injury 4 weeks prior to analyses. PBMCs of each donor were used to reconstitute 4 NSG mice: n = 2 with and n = 2 without (sham) arterial injury. Four weeks after injection, human PBMC engraftment was evaluated in spleen and peripheral blood by flowcytometry.

### Antibodies and flow cytometry

Flowcytometry analysis of human PBMC cell populations in engrafted NSG mice 4 weeks after reconstitution, anti-h(uman)CD3 (clone UCHT1), hCD4 (clone RPA-T4), hCD8 (clone HIT8a), hCD14 (clone MφP9), hCD16 (clone 3G8), hCD45 (clone H130), and anti-m(ouse)CD45 (clone 30-F11) fluorochrome-conjugated mAbs and appropriate isotype controls (all from Biolegend, San Diego, CA, USA) were used. Antibodies were conjugated with fluorescein isothiocyanate (FITC), phycoerythrin (PE), peridinin chlorophyll (PerCP), allophycocyanin (APC), phycoerythrin-Cyanin 5 (PE-Cy5), phycoerythrin-Cyanin 7 (PE-Cy7) or Alexafluor700. Single-cell suspensions of mouse spleen (depleted of erythrocytes) and peripheral whole blood were incubated with anti-CD16/32 (BD Biosciences) for 5 min at 4 °C to block non-specific Fc binding. Antibodies at the appropriate dilutions were incubated with splenocytes or 200 μl whole blood for 30 min at 4 °C. Following red blood cell lysis, blood samples and labeled splenocytes cells were fixed in 1% paraformaldehyde in phosphate-buffered saline. At least 20,000 events were acquired with an LSR II instrument and analyzed using FACS DIVA software (BD Biosciences).

### Measurement of human cytokine levels in NSG mice

The Cytokine Human Ultrasensitive 10-Plex Panel for the Luminex^®^ platform (Invitrogen Corporation, Carlsbad, CA, USA) was carried out to determine the PBMC-engrafted NSG mouse plasma concentrations of human IFNγ, IL-6, IL-4, IL-1β, IL-10, IL-8, GM-CSF, IL-2, IL-5 and TNFα.

### Quantification of neointima formation and medial area

After sacrifice, isolated zinc-fixed carotid and femoral arteries were dehydrated and embedded in paraffin. Serial cross-sections (3 μm) were cut and histomorphometric analysis was performed at 20 cross-section levels (with 66 μm intervals) (Fig. S1).

Sections at each level were stained with Verhoeff-van Gieson or Masson’s trichrome for collagen content and slide images captured using a Hamamatsu Nanozoomer 2.0HT (Hamamatsu Photonics, Hamamatsu, Japan). Morphometric analysis was performed using Aperio Imagescope software (Aperio Technologies inc, CA, USA). Percentage intimal expansion throughout the arterial segment was calculated with the following formula: % intimal expansion = ((A_I_ − A_L_)/A_I_)) × 100; where A_I_ is the area within the internal elastic lamina and A_L_ is the luminal area. The intima/media (I/M) ratio was calculated with the following formula: I/M ratio = (A_I_ − A_L_)/(A_M_ − A_I_); where A_M_ is the area within the external elastic lamina. The media and adventitial areas were also quantified. To determine loss of cells after vascular injury, the total number of cells in media and adventitia were quantified. Serial sections, as depicted in Fig. S1, were quantified to determine the mean % intimal expansion per artery. The number of sections analyzed was an average of 12 (range 9–17) per injured artery and an average of 4 sections (range 3–5) per control artery.

### Detection of human PBMC-derived cells using immunofluorescence

Zinc-fixed paraffin-embedded sections were deparaffinized in xylol, rehydrated in graded ethanol series and demi-water, and then stained using the following antibodies: anti-h-αSMA (clone 1A4), hCD4 (clone 4B12), hCD8 (clone C8/144B), hCD68 (clone KP-1), hCD45 (clone 2B11 + clone PD7/26), vWF (clone F8/86), hCD31 (clone JC70A) (all from DAKO, Glostrup, Denmark) and anti-mouse CD31 (clone MEC 13.3; BD Pharmingen). Sections were first blocked with 3% BSA and subsequently incubated for 1 hour with primary antibodies. Binding of primary antibodies was detected by incubating with the appropriate fluorescently-labeled secondary antibodies: goat anti-mouse IgG1-Alexafluor488 and goat anti-mouse-Alexafluor555 (both from Invitrogen) and goat anti-rabbit IgG-Cy3 (Invitrogen) or rabbit anti-rat biotin (DAKO). Biotinylated antibody complexes were detected with streptavidin-Alexafluor555 (Invitrogen). Sections were mounted in Aqua/polymount (Polysciences Inc., Warrington, PA, USA) containing DAPI (1.5 μg/ml; Invitrogen). Fluorescence microscopy was performed using a Leica DMLB microscope (Leica Microsystems, Rijswijk, The Netherlands) equipped with a Leica DC300F camera and Leica QWin 2.8 software. To confirm antibody specificity, immunohistochemistry was performed on zinc-fixed (NSG spleen with or without human PBMCs) or formalin-fixed (human appendix) paraffin sections. Sections were deparaffinized in xylol, rehydrated in graded ethanol series and demi water, and subsequently stained using immunohistochemistry in a Ventana Benchmark Ultra automated IHC/ISH slide staining system (Ventana Medical Systems, Inc.). For confocal microscopy, sections were deparaffinized in xylol, rehydrated in graded ethanol series and demi water, followed by heat-induced epitope retrieval (HIER) in 10 mM Sodium Citrate Buffer (pH 6.0) for 20 min in a microwave (300 W). The Mouse on Mouse (M.O.M.) Basic Kit (Vector^®^ Laboratories) was used, followed by incubation with primary antibodies (CD45/αSMA and CD8/αSMA) and goat anti-mouse IgG1-Alexafluor488 and goat anti-mouse-Alexafluor555 secondary antibodies as described above. Nuclei were stained with DAPI (Life Technologies) and mounted in Fluorescence Mounting Medium (DAKO). Confocal microscopy was performed using an inverted microscope (Zeiss LSM 780 NLO; Axio Observer Z1).

### Phenotypic characterization of SMCs in sham-operated and injured carotid and femoral arteries

To analyse whether neointimal αSMA + cells are SMCs, immunofluorescent double labeling was performed. Heat-induced epitope retrieval (HIER) was performed in 10 mM Sodium Citrate Buffer (pH 6.0) for 20 min in a microwave (300 W). Sections were blocked with 3% BSA and subsequently incubated for 1 hour with primary antibodies diluted in PBS/1% BSA. The following primary antibodies were used: αSMA (mIgG2a, clone 1A4), SM22α (rabbit polyclonal ab14106, Abcam), and calponin (rabbit polyclonal ab46794, Abcam). Binding of primary antibodies was detected by incubating with fluorescently-labeled secondary antibodies. For αSMA, goat-anti-mouse IgG2a-Alexafluor555 (Invitrogen) or goat-anti-mouse IgG2a-biotin (SouthernBiotech) were used. Biotin was detected with streptavidin-AlexaFluor488 conjugate (Molecular Probes). For SM22α and calponin, goat anti-rabbit IgG-FITC (DAKO) or goat anti-rabbit Cy3 (Zymed Laboratories) were used. Nuclei were stained with DAPI (BioLegend) and mounted in Fluorescence Mounting Medium (DAKO). Images were acquired using TissueFAXS acquisition software (TissueGostics) on a Zeiss Axio Observer Z1 inverted microscope.

### Statistical analysis

Statistical evaluations were performed with Graphpad Prism software (version 5; La Jolla, CA, USA). Data are presented as mean ± SEM. Differences in the means of parametric data were compared by one-way ANOVA with Bonferroni post-tests. Medians of non-parametric data were compared using the Mann–Whitney *U*-test.

## Additional Information

**How to cite this article**: Moser, J. *et al*. Distinct Differences on Neointima Formation in Immunodeficient and Humanized Mice after Carotid or Femoral Arterial Injury. *Sci. Rep.* **6**, 35387; doi: 10.1038/srep35387 (2016).

## Supplementary Material

Supplementary Information
