# Supplementary material for: Distinct Differences on Neointima Formation in Immunodeficient and Humanized Mice after Carotid or Femoral Arterial Injury
Source: Sci Rep. 2016 Oct 19;6:35387. doi: 10.1038/srep35387 (PMC5069488; doi:10.1038/srep35387)
Supplement: Supplementary Information [file srep35387-s1.pdf]

SUPPLEMENTAL INFORMATION

DISTINCT DIFFERENCES ON NEOINTIMA FORMATION IN IMMUNODEFICIENT AND HUMANIZED MICE AFTER CAROTID OR FEMORAL ARTERIAL INJURY

Jill Moser[1,5], Joris van Ark[1], Marcory C. van Dijk[2], Dale L. Greiner[3], Leonard D. Shultz[4], Harry van Goor[1], and Jan-Luuk Hillebrands[1,*]

*** Corresponding author:**

J.L. Hillebrands, PhD

Department of Medical Biology and Pathology- Pathology section

University of Groningen, University Medical Center Groningen

Hanzeplein 1, PO Box 30.001

Groningen, The Netherlands.

Email: j.l.hillebrands@umcg.nl

Tel: +31-50-3612875

Fax: +31-50-3619107

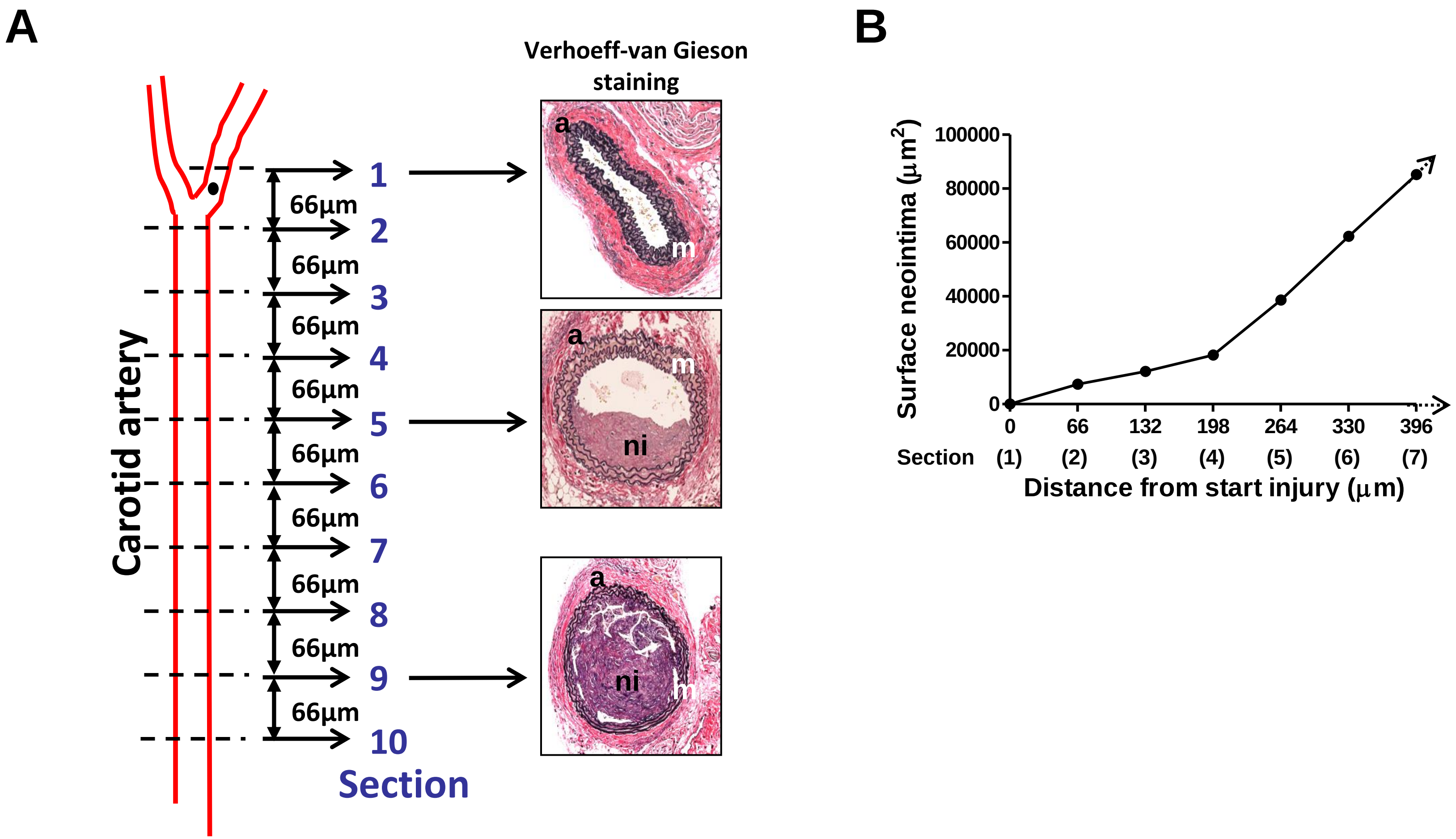

Figure S1. Schematic representation of carotid artery wire-injury and tissue microtomy. (A) Intimal expansion was measured at 20 cross-section levels (66 µm intervals) of the sham-operated and the wire-injured carotid artery 4 weeks after injury (with or without human PBMC reconstitution). Verhoeff-van Gieson staining was performed to visualize elastic laminae. Abbreviations: a: adventitia; m: media; ni: neointima. (B) Typical example of the increase in surface neointima throughout the carotid artery beginning just before the start of injury (section 1). Original magnification photomicrographs: 200x

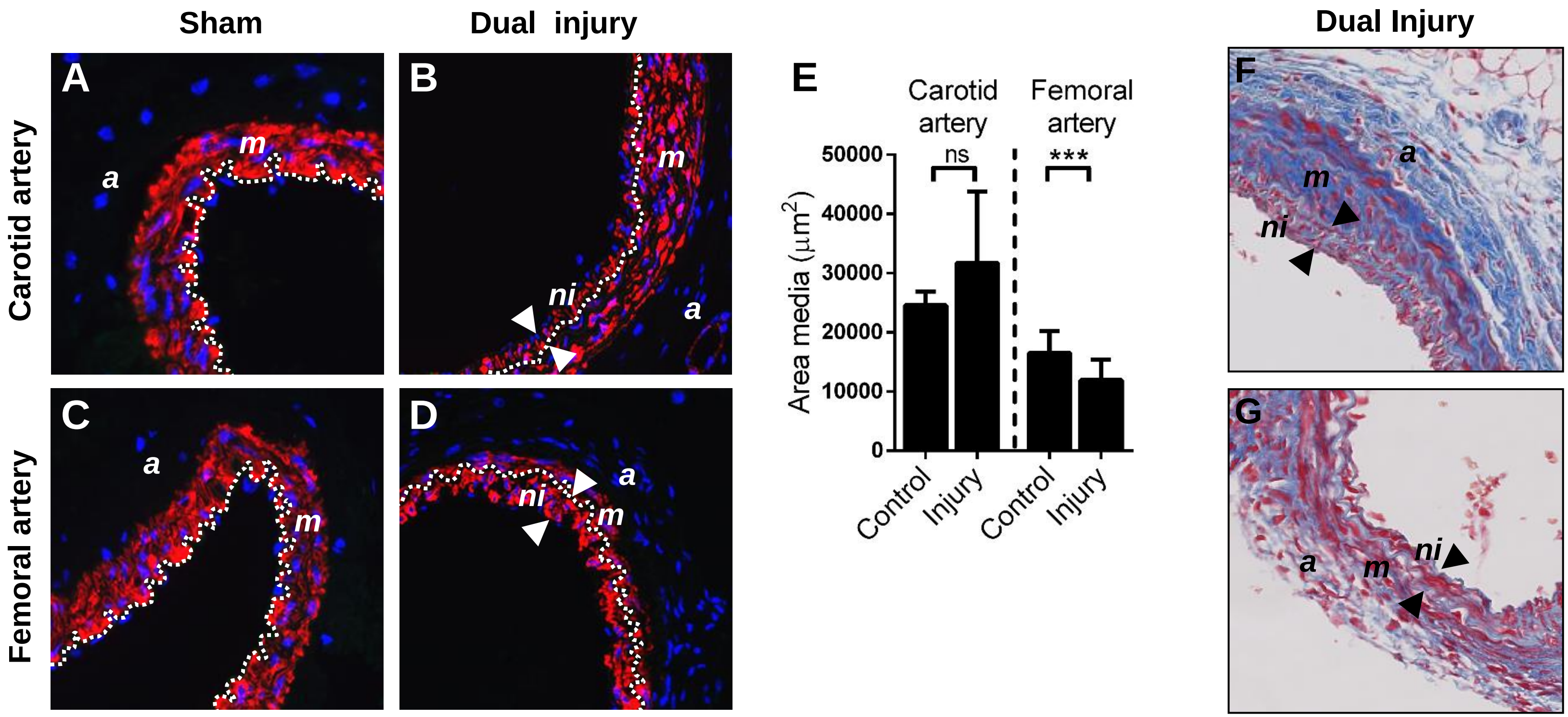

Figure S2. Neointima formation 4 weeks after dual vascular injury in BL/6 control mice. Representative sections of carotid (A,B) and femoral (C,D) arteries from sham-operated and dual injured mice stained for αSMA (red). (A) Sham-operated control carotid artery, (B) Carotid artery after dual injury, (C) Sham-operated control femoral artery, (D) Femoral artery after dual injury. Nuclei were stained with DAPI (blue), (E) Area of the media (μm^2), Representive sections of dual injured arteries stained with Masson's trichrome stain, (F) carotid artery and (G) femoral artery after dual injury. Neointima is indicated as tissue in between arrowheads. Dotted lines indicate the internal elastic lamina. Abbreviations: *a*: adventitia; *m*: media; *ni*: neointima. Original magnification: 400x. (***P<0.001)

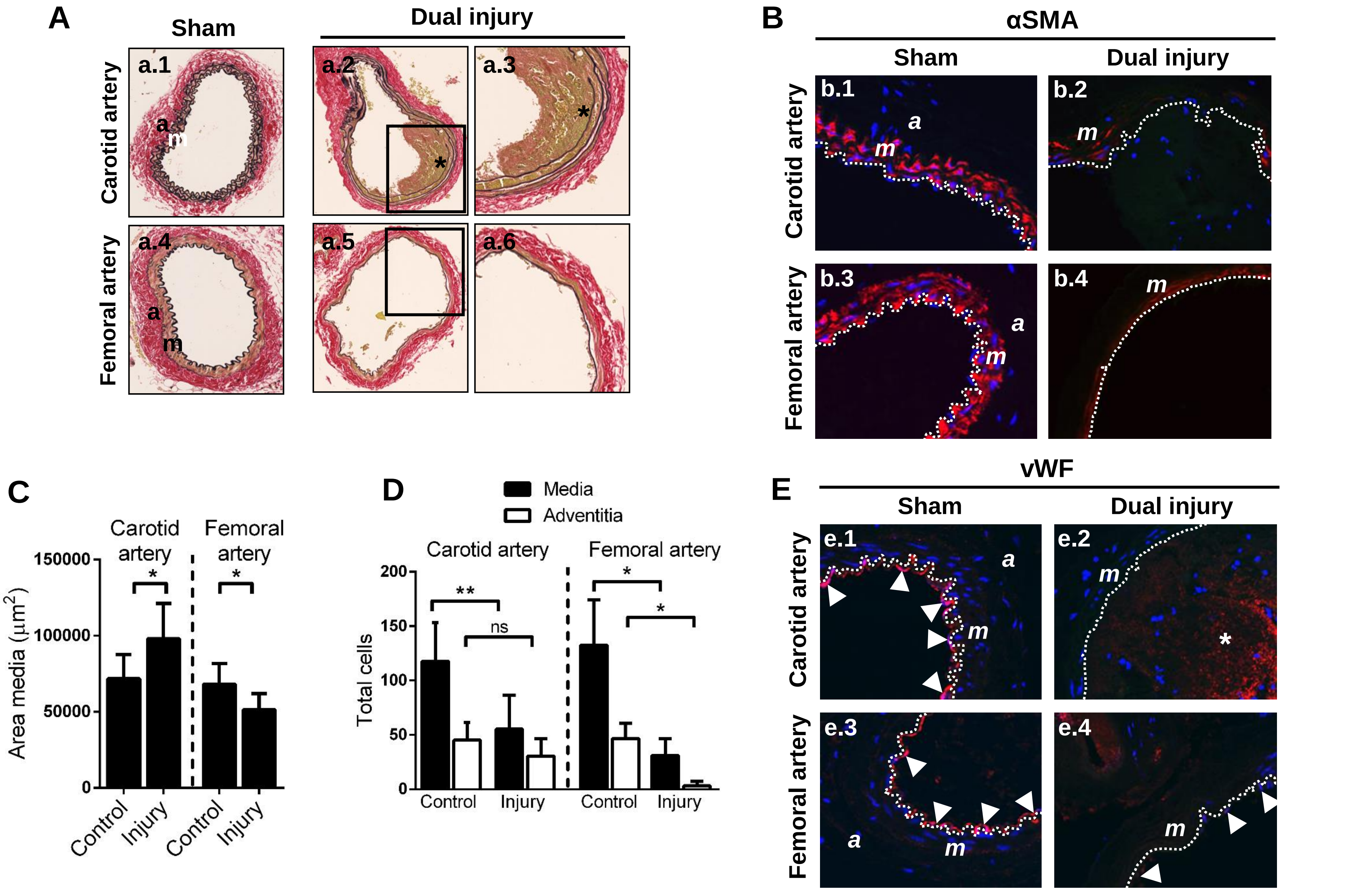

Figure S3. Early response to dual vascular injury in NSG mice. (A) Representative sections of carotid and femoral arteries from dual injured and sham-operated mice stained with Verhoeff-van Gieson. a.3 and a.6 are higher power magnifications of the framed regions depicted in a.2 and a.5, respectively. (B) Immunofluorescent staining for αSMA (red). Area of the media (µm^2), n=4 mice (D) The total number of cells present in the media and adventitia of the carotid and femoral artery after dual injury, n=4 mice (D) Immunofluorescent staining for vWF (red). Nuclei were stained with DAPI (blue). Dotted lines indicate the internal elastic lamina. Asterisks indicate thrombi. Arrowheads indicate vWF$^+$ endothelial cells. Abbreviations: *a*: adventitia; *m*: media. Original magnification: A: 200x; B & D: 400x (** P<0.01, *P<0.05)

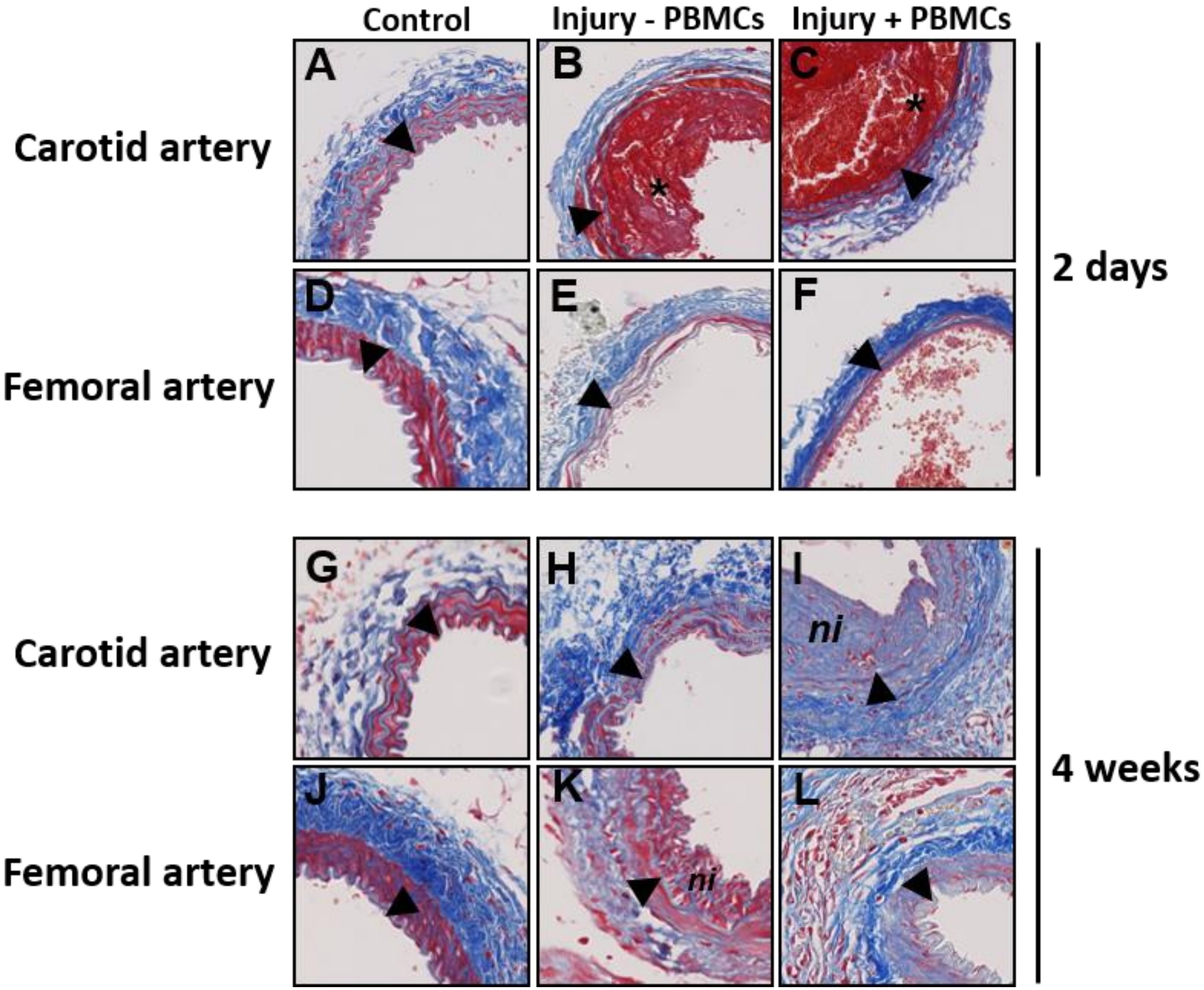

Figure S4. Masson's trichrome analyses of sham and injured arteries in NSG mice. Representative sections of carotid (A-C) and femoral arteries (D-F) 2 days after dual injury or sham-operated mice, and carotid (G-I) and femoral arteries (J-L) 4 weeks after dual injury or sham operated mice. (B, E, H &I) represent injured arteries in immunodeficient mice and (C, F, I & L) represent injured arteries from mice which were reconstituted with hPBMCs. Arrowheads indicate the internal elastic lamina. Asterisks indicate thrombi. Abbreviations: *a*: adventitia; *m*: media; *ni*: neointima. Magnification: A: 400x.

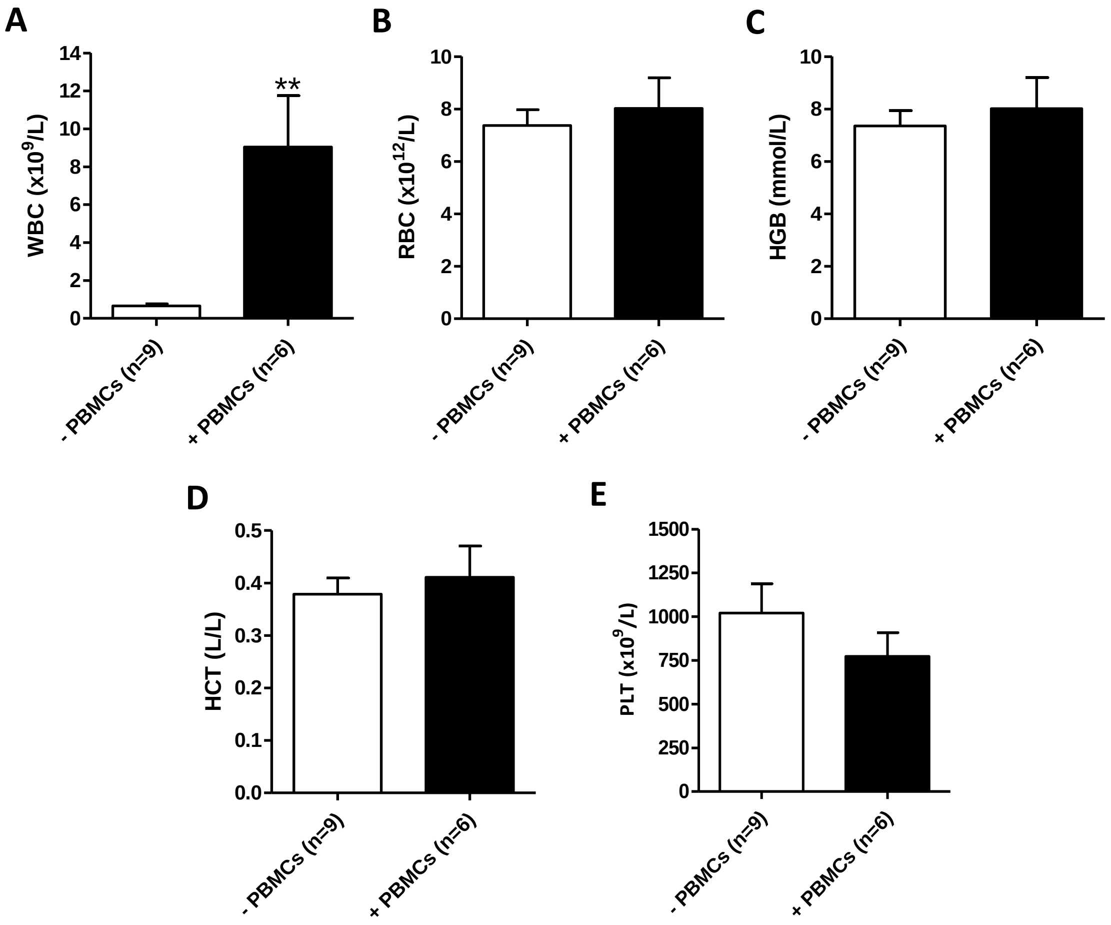

Figure S5. Haematological analyses of NSG mice 4 weeks after reconstitution with 5x10^6 human PBMCs (black bars) or without reconstitution (white bars). (A) white blood cell (WBC) counts, (B) red blood cell counts (RBC), (C) haemoglobin (HGB), (D) haematocrit (HCT), and (E) platelet (PLT) counts. The n-value reflects the number of mice included. Data are expressed as mean±SEM, **P<0.01.

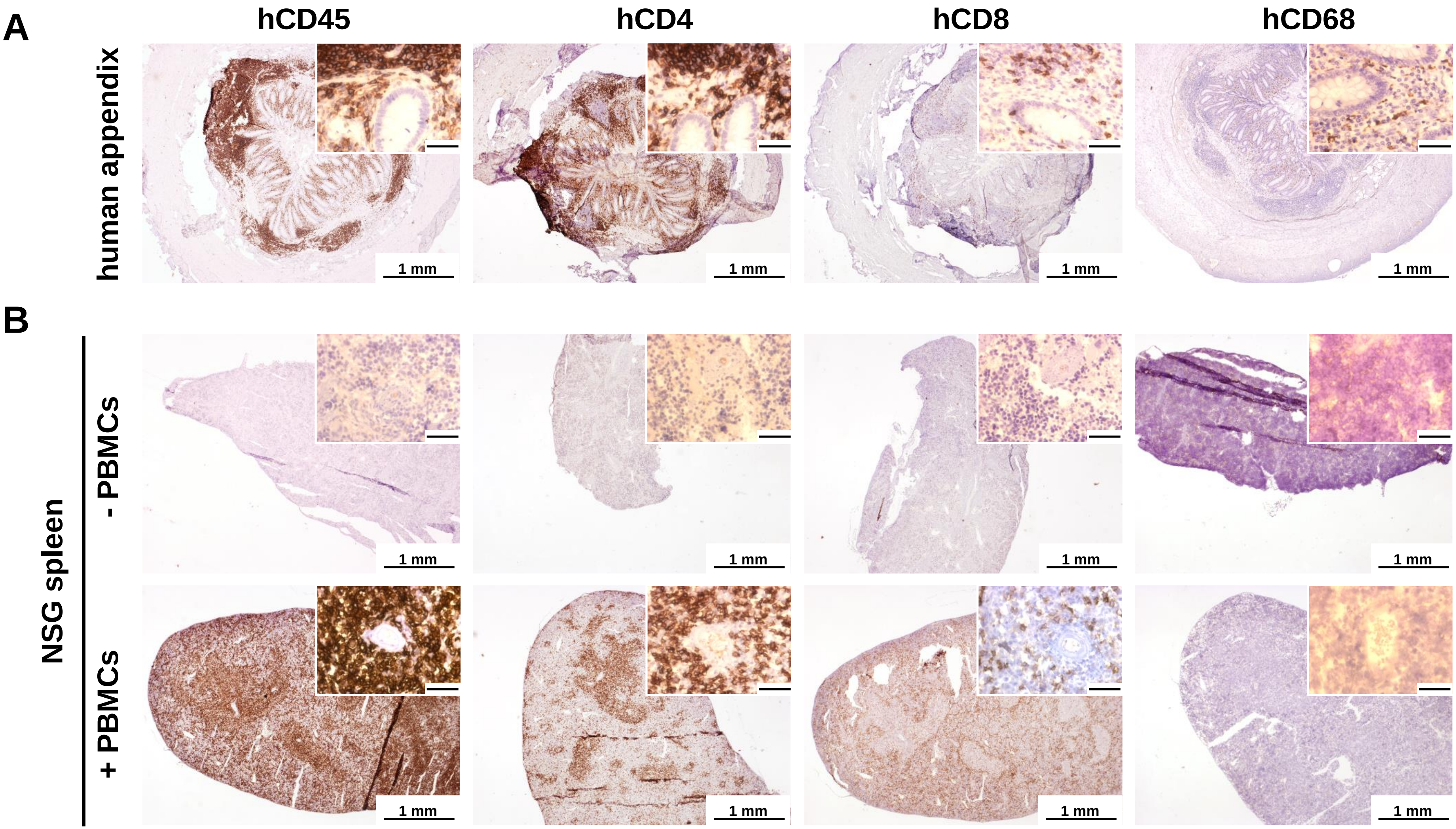

Figure S6. Confirmation of the specificity of human-specific antibodies on human appendix and NSG spleen using immunohistochemistry. (A) Representative sections of human appendix stained for hCD45, hCD4, hCD8 and hCD68. These stainings were used as positive control and revealed positive cells in the submucosa and lamina propria. (B) Spleen from NSG mice without (upper row) or with (bottom row) human PBMC reconstitution and analyzed 4 weeks after cell transfer. Human PBMC-derived cells ($hCD45^+$) were only detected in spleens from NSG mice that were reconstituted with human PBMCs. Engrafted human cells were $CD4^+$ or $CD8^+$; no human $CD68^+$ macrophages were detected. Sections were stained using immunohistochemistry in a Ventana Benchmark Ultra automated IHC/ISH slide staining system. Original magnification: 20x (insets: 400x). Scale bar inset: 50 µm.

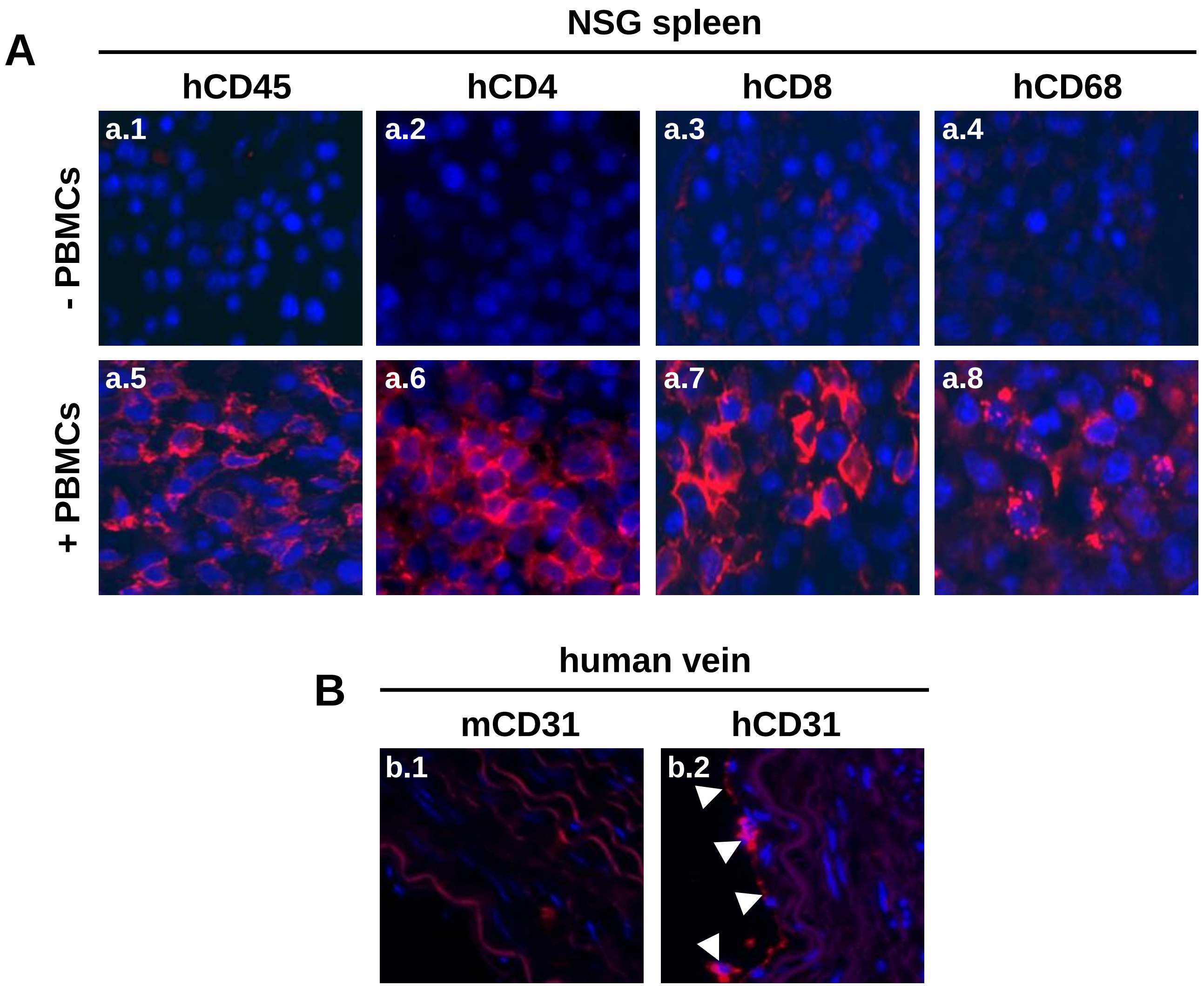

Figure S7. Confirmation of the specificity of human-specific antibodies on NSG spleen and human vein. (A) Representative sections of spleen from NSG mice without (a.1-4) or with (a.5-8) human PBMC reconstitution 4 weeks after cell transfer. Sections were immunofluorescently labeled with human-specific antibodies recognizing human CD45 (a.1 & a.5), human CD4 (a.2 & a.6), human CD8 (a.3 & a.7), and human CD68 (a.4 & a.8). Human PBMC-derived cells were only detected in spleens from NSG mice that were reconstituted with human PBMCs. (B) Endothelial cells in human vein were only detected using hCD31 (arrowheads, b.2) and not mCD31 (b.1) antibody. Nuclei were stained with DAPI (blue). Original magnification: 400x
